# Gender Difference in the Association Between Metabolic Factors and Hepatocellular Carcinoma

**DOI:** 10.1093/jncics/pkaa036

**Published:** 2020-05-08

**Authors:** Chi-Ling Chen, Ming-Jeng Kuo, Amy Ming-Fang Yen, Wei-Shiung Yang, Jia-Horng Kao, Pei-Jer Chen, Hsiu-Hsi Chen

**Affiliations:** p1 Graduate Institute of Clinical Medicine, College of Medicine, National Taiwan University, Taipei, Taiwan; p2 Graduate Institute of Epidemiology and Preventive Medicine, College of Public Health, National Taiwan University, Taipei, Taiwan; p3 Department of Surgery, National Taiwan University Hospital, Taipei, Taiwan; p4 Department of Hepatogastroenterology, Tainan Municipal Hospital, Tainan, Taiwan; p5 School of Oral Hygiene, College of Oral Medicine, Taipei Medical University, Taipei, Taiwan; p6 Department of Internal Medicine, National Taiwan University Hospital, Taipei, Taiwan; p7 Hepatitis Research Center, National Taiwan University Hospital, Taipei, Taiwan

## Abstract

**Background:**

A gender difference in hepatocellular carcinoma (HCC) that men have higher incidence than women has long been noted and can be explained by the cross-talk between sex hormones and hepatitis B virus/hepatitis C virus (HBV/HCV). Whether metabolic factors yield similar sexual difference in non-HBV/HCV-HCC remains elusive.

**Methods:**

There were 74 782 hepatitis B surface antigen (HBsAg)/antibody to hepatitis C virus (anti-HCV) negative residents who participated in the Keelung Community-Based Integrated Screening program and were followed in 2000-2007. Incident HCC was identified by linkage to the Taiwan Cancer Registry. Cox proportional hazards regression models were used to estimate the association between metabolic factors and HCC stratified by sex. All statistical tests were 2-sided.

**Results:**

With 320 829 follow-up person-years, 99 residents developed HCC. The adjusted hazard ratios (aHR) were 2.10 (95% confidence interval [CI] = 1.07 to 4.13) and 3.71 (95% CI = 2.01 to 6.86) for prediabetes and diabetes, respectively, in men. The corresponding adjusted hazard ratios were 1.16 (95% CI = 0.48 to 2.83) and 1.47 (95% CI = 0.65 to 3.34) in women. Compared with normal weight (body mass index [BMI] = 23-25), underweight (BMI < 21, HR = 3.56, 95% CI = 1.18 to 10.8) and overweight (BMI = 25 to <27.3, HR = 3.81, 95% CI = 1.43 to 10.2) were associated with an elevated risk in men. The statistically significant gradient relationship per advanced BMI category was noted in women (aHR = 1.41, 95% CI = 1.07 to 1.87). The HCC–fasting glucose (*P* = .046) and HCC-BMI (*P* = .03) associations were statistically significantly modified by sex. Elevated aspartate aminotransferase, aspartate aminotransferase-to-platelet index and fibrosis index, and habitual alcohol consumption were related to HCC only in men, whereas increased alanine aminotransferase and lower platelet levels predicted HCC risk in women.

**Conclusions:**

We found that BMI-HCC associations were U-shape for men and linear for women, and the elevated HCC risk began from glucose impairment in men only. Whether good glycemic and weight control can reduce HCC risk warrants further investigation.

Liver cancer (85%-90% hepatocellular carcinoma) ranks sixth in neoplasm and second in cancer death worldwide (1). Chronic viral hepatitis (hepatitis B virus [HBV] or hepatitis C virus [HCV]) and excessive alcohol consumption are well-known risk factors for hepatocellular carcinoma (HCC). Components of metabolic syndrome including obesity and diabetes were reported as an independent risk factor for HCC in many countries with various HB/HC prevalence (2-4). The mass vaccination of hepatitis B and effective treatment of hepatitis C should substantially reduce the incidence and mortality of HCC when the majority of the population had been vaccinated or treated. However, the incidence of HCC is still increasing over the past decades in most countries (5), which is commensurate with the increased prevalence of obesity and diabetes.

Sex disparity has been observed in HCC of male predominance with a 2-5 times higher incidence, regardless of the etiology of HCC (5). The sex disparity in HBV/HCV-related HCC can be explained by the cross-talk between sex hormone and HBV/HCV (6,7). Animal and human studies indicated that sex disparity may originate from different sexual hormones serving different roles: androgen as tumor promoter and estrogen as tumor suppressor (8-11). Although some individual studies reported higher HCC risk in diabetic men, a recent meta-analysis showed a nonsignificant, slightly higher HCC risk in diabetic men than women (standardized rate ratio [SRR] = 1.96, 95% confidence interval [CI] = 1.71 to 2.24 vs SRR = 1.66, 95% CI = 1.14 to 2.41, respectively; *P* = .41 for gender difference) (3). Two meta-analyses demonstrated that sex disparity exists in the obesity and HCC association, in that obese men had higher risk of HCC than obese women (12,13). A nonlinear dose-response trend was noted for men, and the gradient increase was faster in men than women (13). Yao et al. also reported that sex difference was more pronounced in non-Asian studies (13). The similarity between men and women in Asian studies may result from the high prevalence of HBV/HCV infections, which contributed to the most HCC cases in Asia. Nevertheless, further research is still needed to clarify the sex disparity in metabolic factors and HCC association without the influence of HBV/HCV.

We conducted current analysis to elucidate the relationship between metabolic factors including diabetes, obesity, and dyslipidemia and HCC risk among HBV/HCV seronegatives in a community-based cohort with emphasis on sex disparity. We further explored whether the HCC risk started to increase at prediabetic stage and demonstrated the important predictors for HCC in men and women separately.

## Methods

### Study Cohort

The study cohort consisted of participants in the Keelung-Community-Based Integrated Screening program in Taiwan; 212 954 residents aged 30 years and older were invited to participate in a multiple screening program, which was launched in 2000 and aims to provide periodic screenings for a variety of cancers and chronic diseases (14). Those who tested positive in hepatitis B surface antigen (HBsAg) or antibody to hepatitis C virus (anti-HCV) were excluded, resulting in a total of 74 782 residents (46 248 female and 28 534 male) remaining in this analysis. All participants provided informed consent, and the study was approved by the institutional review board of the Chang Gung University.

### HCC Confirmation

Mass screening for HCC was carried out in a 2-stage approach in the Keelung-Community-Based Integrated screening program, and the detailed confirmation process is described elsewhere (14). Subjects suspected of malignant nodular lesions were referred to further confirmatory diagnosis. All HCC cases in our analysis met one of the following criteria: histopathological proof, detection by at least 2 image tools (computed tomography, angiography, or magnetic resonance image), and 1 imaging diagnosis plus serum alpha fetoprotein level greater than 400 ng/mL. To ensure complete identification of HCC incident cases, linkage using participants’ unique national identification numbers to the Taiwan Cancer Registry Profile was conducted. After excluding prevalent cases, a total of 99 HCC cases were identified.

### Data Collection

Demographic and lifestyle information, including habitual smoking and alcohol intake status, was collected via questionnaire by physicians or registered nurses in a standardized manner at enrollment. Fasting blood samples were also collected and tested on serum HBsAg, anti-HCV using commercial kits by the ELISA method (14). Body mass index (BMI) was measured at baseline and was categorized into less than 21, 21-23, 23-25, 25 to less than 27.3, and no less than 27.3 kg/m^2^. Central obesity was defined as waist circumferences greater than 90 cm for men and greater than 80 cm for women. Serum triglyceride (TG) at least 150 mg/dL and cholesterol at least 240 mg/dL were considered hypertriglycemia and hypercholesterolemia, respectively. High-density lipoprotein (HDL) cholesterol was categorized as low (<40 mg/dl in men and <50 mg/dl in women) and normal, and low-density lipoprotein (LDL) cholesterol into 2 categories at 130 mg/dL(<130 vs ≥130 mg/dL). Quintiles were used to categorize systolic blood pressure (SBP), diastolic blood pressure (DBP), TG, total cholesterol (TC), HDL, and LDL when linear trend was the interest. Metabolic syndrome (yes/no) and metabolic score defined by the number of metabolic factors included in metabolic syndrome were examined. Two widely used noninvasive indices for significant liver fibrosis—aspartate aminotransferase-to-platelet index (APRI) and fibrosis index (FIB-4)—were calculated, which were derived from aspartate aminotransferase (AST), platelet, age, and alanine aminotransferase (ALT). Subjects who identified as being diabetic or on antihyperglycaemic treatment at the baseline questionnaire or those with glucose levels higher than 126 mg/dL were considered diabetics. Participants with fasting glucose levels between 100 and 125 mg/dL were defined as prediabetic.

### Statistical Analysis

Person-years for each subject were calculated from the dates of enrollment to the dates of HCC diagnosis, deaths, or December 31, 2007, whichever came first. Those who were alive and HCC-free were censored on December 31, 2007. Nelson-Aalen cumulative hazard method was used to estimate the cumulative incidence of HCC, and the log-rank test was conducted to compare incidence between different groups. Cox proportional hazards regression models were conducted to estimate the hazard ratios (HRs) and 95% confidence intervals for HCC. All models were adjusted for age, and other metabolic factors and relevant prespecified HCC risk factors were included in the multivariable models. The proportional hazards assumption was examined by the Schoenfeld residuals method, and all main metabolic factors met the requirement. The estimated results for other variables were based on the model with categorized fasting blood sugar (normal, prediabetes, and diabetes). To avoid collinearity, the adjusted hazard ratios (aHRs) for fasting glucose in interval scale, metabolic syndrome (no, yes), and metabolic score (0-5) were separately modeled from that with categorized status of fasting blood sugar (normal, prediabetes, and diabetes). The adjusted hazard ratios for BMI categories were separately modeled from that with BMI categories in group linear form. Stepwise regression models were performed to identify important HCC risk factors separately for men and women. The variable selection was based on the model with categorized fasting blood sugar (normal, prediabetes, and diabetes) with *P* value equal to  .05 as model entry and removal criteria. To avoid multiple testing while considering continuous or categorical property of variables pertaining to metabolic factors, we analyzed a series of models like fasting glucose per 10 mg/dL increment, metabolic syndrome (no, yes), and metabolic score.

For men, the stepwise model selection process with categorized fasting blood sugar (normal, prediabetes, and diabetes) as the main metabolic factor resulted in age, serum AST level, APRI index, and habitual alcohol consumption in the final model. All other selected metabolic factors, including metabolic syndrome, metabolic score, diabetes categories, fasting glucose per 10 mg/dl increment, and BMI categories, were modeled separately with the aforementioned variables. For women, the stepwise model selection process with categorized fasting blood sugar (normal, prediabetes, and diabetes) as main metabolic factor resulted in age, serum AST level, and serum platelet level in the final model. All other selected metabolic factors including metabolic syndrome, metabolic score, diabetes categories, fasting glucose per 10 mg/dl increment, and BMI per advanced group were modeled separately with the aforementioned variables. Linear trends were tested by modeling an ordinal variable indicating the median value of each exposure category, with *P* values less than .05 considered statistically significant on the basis of Wald test. The presence of effect modification (ie, a different relationship between metabolic factors and HCC between men and women) was tested by an interaction term between these factors and sex. This *P* value derived from Wald test for interaction term examined a departure from a multiplicative relation. All analysis was conducted with SAS version 9.3. All statistical tests were 2-sided.

## Results

### Cumulative Incidence of HCC and Baseline Characteristics by Sex

**Figure 1.**
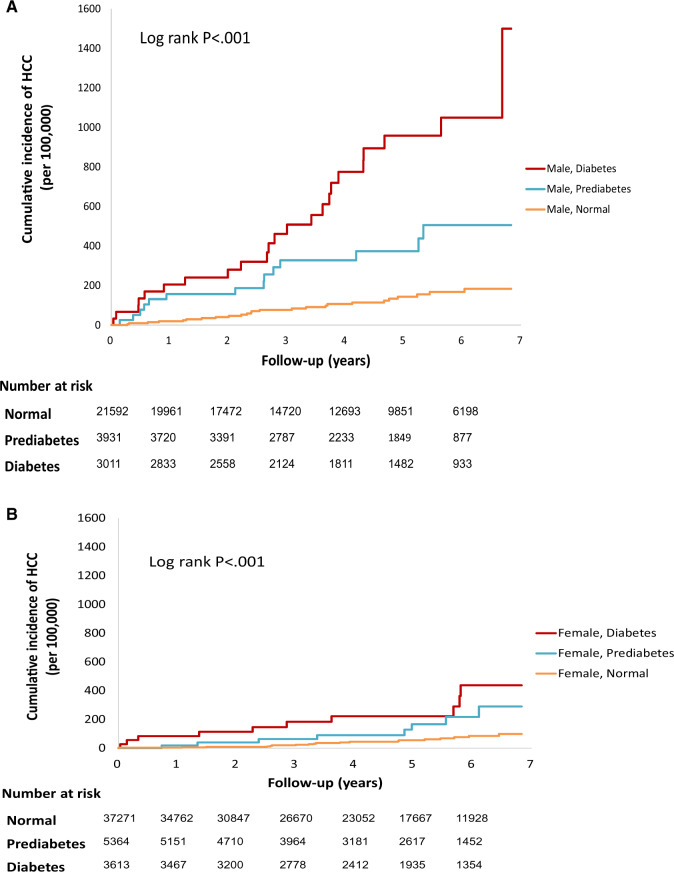
Cumulative hazard estimate of the incident hepatocellular carcinoma and fasting glucose stratified gender in the HBV/HCV seronegative community cohort from 1999 to 2007 in Keelung, Taiwan. A) Male. B) Female. HCC = hepatocellular carcinoma; HBV/HCV = hepatitis B virus/hepatitis C virus.

The median follow-up time was 4.47 years. There were 99 newly diagnosed HCC patients (37 female and 62 male) with 320 829 (201 081 for women and 119 747 for men) person-years of follow-up, yielding an incidence of 30.9 (18.4 for women and 51.8 for men) per 10^5^ person-years. The median time to incident HCC diagnosis was 2.79 years. Figure 1 shows cumulative incidence rates of HCC for different diabetic status stratified by sex. The HCC risk increased from normal, prediabetes to diabetes in a dose-response fashion only in men.

Compared with female participants, male participants were older, attained higher education levels, and were more likely to consume cigarettes and drink higher amounts of alcohol (Table 1). Men also had higher BMI, higher prevalence of metabolic syndrome, prediabetes and diabetes, high blood pressure, and hypertriglyceridemia. Although the TC levels were similar between men and women, women tended to have higher proportion of low HDL levels. Men also had higher proportions of abnormal serum AST (>45 IU/L), ALT (>45 IU/L), and low platelet levels (<150 × 10^9^/L), thus leading to higher fibrosis scores.

**Table 1.** Baseline distribution of selected metabolic factors and HCC risk factors stratified by gender, Keelung-Community-Based Integrated screening cohort, 2001-2007

Characteristics	Male (n = 28 534)		Female (n = 46 248)		Total (n = 74 782)	
	No. (%)	HCC No.	No. (%)	HCC No.	No. (%)	HCC No.
		(n = 62)		(n = 37)		(n = 99)
Age at recruitment, y						
<40	7513 (26.3)	0	14 765 (31.9)	0	22 278 (29.8)	0
40–49	6285 (22.0)	3	11 635 (25.2)	1	17 920 (24.0)	4
50–59	5135 (18.0)	0	9237 (20.0)	1	14 372 (19.2)	1
60–69	4587 (16.1)	6	6377 (13.8)	7	10 964 (14.7)	13
70–79	3932 (13.8)	23	3448 (7.5)	15	7380 (9.9)	38
>80	1082 (3.8)	30	786 (1.7)	13	1868 (2.5)	43
Mean (SD)	51.5 (16.4)		47.8 (14.8)		49.2 (15.6)	
Education						
>9 years	20 262 (71.0)	21	29 023 (62.8)	4	49 285 (65.9)	25
<9 years	5838 (20.5)	23	10 587 (22.9)	14	16 425 (22.0)	37
No formal education	2214 (7.8)	16	6306 (13.6)	18	8520 (11.4)	34
Missing	220 (0.8)	2	338 (0.7)	1	558 (0.7)	3
Marital status						
Unmarried	4261 (14.9)	2	5918 (12.8)	0	10 179 (13.6)	2
Married	22 055 (77.3)	53	32 137 (69.5)	19	54 192 (72.5)	72
Divorced/widowed	2216 (7.8)	7	8188 (17.7)	18	10 404 (13.9)	25
Missing	2 (0.01)	0	5 (0.01)	0	7 (0.01)	0
Cigarette smoking at enrollment^a^						
Never	13 864 (48.6)	30	42 524 (91.9)	34	56 388 (75.4)	64
Low	5994 (21.0)	5	2918 (6.3)	3	8912 (11.9)	8
High	8676 (30.4)	27	806 (1.7)	0	9482 (12.7)	27
Habitual alcohol consumption^a^						
Never	21 658 (75.9)	48	44 266 (95.7)	37	65 924 (88.2)	85
Low	3007 (10.5)	3	1405 (3.0)	0	4412 (5.9)	3
High	3869 (13.6)	11	577 (1.2)	0	4446 (5.9)	11
Metabolic syndrome						
No	21 153 (74.1)	35	36 832 (79.6)	18	57 985 (77.5)	53
Yes	6869 (24.1)	27	8626 (18.7)	17	15 495 (20.7)	44
Missing	512 (1.8)	0	790 (1.7)	2	1302 (1.7)	2
Central obesity						
No	20 496 (71.8)	39	33 331 (72.1)	10	53 827 (72.0)	49
Yes	7673 (26.9)	23	12 367 (26.7)	25	20 040 (26.8)	48
Missing	365 (1.3)	0	550 (1.2)	2	915 (1.2)	2
BMI (kg/m^2^)						
<21	3505 (12.3)	9	11 333 (24.5)	2	14 838 (19.8)	11
21 to <23	4897 (17.2)	11	9952 (21.5)	2	14 849 (19.9)	13
23 to <25	6371 (22.3)	6	8474 (18.3)	7	14 845 (19.9)	13
25 to <27.3	7031 (24.6)	21	7784 (16.8)	9	14 815 (19.8)	30
>27.3	6477 (22.7)	15	8373 (18.1)	16	14 850 (19.9)	31
Missing	253 (0.9)	0	332 (0.7)	1	585 (0.8)	1
Mean (SD)	24.9 (3.5)		23.9 (4.0)		24.3 (3.9)	
Fasting glucose						
Normal	21 592 (75.7)	25	37 271 (80.6)	19	58 863 (78.7)	44
Prediabetes	3931 (13.8)	14	5364 (11.6)	8	9295 (12.4)	22
Diabetes	3011 (10.6)	23	3613 (7.8)	10	6624 (8.9)	33
Blood pressure						
<130/85	12 259 (43.0)	15	29 155 (63.0)	7	41 414 (55.4)	22
>130/85	16 047 (56.2)	47	16 747 (36.2)	30	32 794 (43.9)	77
Missing	228 (0.8)	0	346 (0.7)	0	574 (0.8)	0
Serum TG level (mg/dl)						
<150	18 032 (63.2)	43	36 255 (78.4)	24	54 287 (72.6)	67
>150	10 502 (36.8)	19	9993 (21.6)	13	20 495 (27.4)	32
Mean (SD)	158.8 (146.3)		117.5 (103.9)		133.2 (123.5)	
Serum total cholesterol (mg/dl)						
<240	24 959 (87.5)	55	39 830 (86.1)	28	64 789 (86.6)	83
>240	3575 (12.5)	7	6418 (13.9)	9	9993 (13.4)	16
Mean (SD)	197.2 (38.4)		197.4 (39.7)		197.3 (39.2)	
HDL (mg/dl) ^b^						
Normal	23 696 (83.0)	51	36 002 (77.8)	25	59 698 (79.8)	76
Low	4838 (17.0)	11	10 244 (22.2)	12	15 082 (20.2)	23
Missing	0 (0.0)	0	2 (0.0)	0	2 (0.0)	0
Mean (SD)	51.5 (13.8)		60.6 (13.9)		57.1 (14.6)	
LDL (mg/dl)						
<130	19 246 (67.4)	45	32 656 (70.6)	23	51 902 (69.4)	68
>130	8937 (31.3)	15	13 208 (28.6)	14	22 145 (29.6)	29
Missing	351 (1.2)	2	384 (0.8)	0	735 (1.0)	2
Mean (SD)	115.7 (33.6)		113.7 (33.7)		114.5 (33.7)	
Serum platelet (10^9^/L)						
>150	26 565 (93.1)	44	44 219 (95.6)	26	70 784 (94.7)	70
<150	1689 (5.9)	18	1570 (3.4)	11	3259 (4.4)	29
Missing	280 (1.0)	0	459 (1.0)	0	739 (1.0)	0
Mean (SD)	228.3 (57.5)		246.4 (62.8)		239.5 (61.4)	
Serum aspartate aminotransferase (IU/L)						
<45	27 543 (96.5)	48	45 117 (97.6)	31	72 660 (97.2)	79
>45	991 (3.5)	14	1131 (2.4)	6	2122 (2.8)	20
Mean (SD)	23.2 (15.2)		20.2 (11.5)		21.3 (13.1)	
Serum alanine aminotransferase (IU/L)						
<45	25 470 (89.3)	48	43 992 (95.1)	28	69 462 (92.9)	76
>45	3064 (10.7)	14	2255 (4.9)	9	5319 (7.1)	23
Missing	0 (0.0)	0	1 (0.0)	0	1 (0.0)	0
Mean (SD)	26.7 (24.5)		19.5 (17.9)		22.3 (21.0)	
APRI index						
<0.38	24 455 (85.7)	27	42 508 (91.9)	20	66 963 (89.5)	47
>0.38	3799 (13.3)	35	3281 (7.1)	17	7080 (9.5)	52
Missing	280 (1.0)	0	459 (1.0)	0	739 (1.0)	0
Mean (SD)	0.28 (0.38)		0.23 (0.56)		0.25 (0.50)	
FIB-4 index						
<1.45	20 778 (72.8)	12	37 908 (82.0)	12	58 686 (78.5)	24
>1.45	7476 (26.2)	50	7880 (17.0)	25	15 356 (20.5)	75
Missing	280 (1.0)	0	460 (1.0)	0	740 (1.0)	0
Mean (SD)	1.21 (1.28)		1.06 (2.52)		1.12 (2.14)	
Family history of HCC						
No	27 206 (95.3)	61	43 853 (94.8)	36	71 059 (95.0)	97
Yes	1328 (4.7)	1	2395 (5.2)	1	3723 (5.0)	2

a The median values were used to split the 3 lifestyle variables into low and high categories. The median value for cumulative consumption of cigarettes, cumulative consumption of alcohol, and cumulative consumption of betel nuts were 5840 pack-days, 3530 glass-weeks, and 3100 quid-days, respectively. APRI = aspartate aminotransferase to platelet index; BMI = body mass index; FIB-4 = fibrosis index; HCC = hepatocellular carcinoma; HDL = high-density lipoprotein; LDL = low-density lipoprotein; TG = serum triglyceride.

b HDL <40 mg/dl in men and <50 mg/dl in women.

### Metabolic Syndrome and Metabolic Score

The hazard ratios of metabolic factors in relation to HCC after adjustment for other metabolic and lifestyle factors are shown in Table 2. Males with metabolic syndrome have a more than 2-fold increased HCC risk (aHR = 2.58, 95% CI = 1.22 to 5.48) and an almost 2-fold higher risk with the addition of 1 risk factor in the component of metabolic syndrome (aHR = 1.77, 95% CI = 1.16 to 2.68) (Table 2), but none of these factors were associated with HCC in women.

**Table 2.** Multivariable analysis of metabolic factors associated with non-HBV/HCV–related HCC after adjusting for other relevant factors

Variables	Male	Female	*P*_interaction_ ^a^
	HR (95% CI)	HR (95% CI)	
Metabolic factors		
Metabolic syndrome^b^	2.58 (1.22 to 5.48)	0.84 (0.32 to 2.21)	0.75
Metabolic score^c^	1.77 (1.16 to 2.68)	1.37 (0.80 to 2.35)	0.38
Diabetes mellitus			0.40
Normal	1.00 (Referent)	1.00 (Referent)	
Prediabetes	2.10 (1.07 to 4.13)	1.16 (0.48 to 2.83)	
Diabetes	3.71 (2.01 to 6.86)	1.47 (0.65 to 3.34)	
*P*_trend_	<0.001	0.34	
Fasting glucose per 10 mg/dL increment^d^	1.11 (1.07 to 1.15)	1.03 (0.97 to 1.10)	0.046
BMI (kg/m^2^)^e^			0.03
<21	3.56 (1.18 to 10.8)	—	^f^
21 to <23	2.56 (0.87 to 7.54)	1.00 (Referent)	
23 to <25	1.00 (Referent)	1.86 (0.58 to 5.93)	
25 to <27.3	3.81 (1.43 to 10.2)	2.24 (0.74 to 6.83)	
>27.3	2.18 (0.78 to 6.11)	2.48 (0.86 to 7.12)	
*P*_trend_	0.80	0.06	
BMI, per advanced group^e^	0.97 (0.79 to 1.20)	1.36 (0.99 to 1.87)	0.05
Blood pressure			0.21
<130/85	1.00 (Referent)	1.00 (Referent)	
>130/85	1.08 (0.58 to 2.04)	2.32 (0.97 to 5.56)	
Serum TG level (mg/dL)			0.75
<150	1.00 (Referent)	1.00 (Referent)	
>150	0.56 (0.30 to 1.06)	0.70 (0.33 to 1.47)	
Serum total cholesterol(mg/dL)			0.59
<240	1.00 (Referent)	1.00 (Referent)	
>240	1.28 (0.49 to 3.32)	1.42 (0.50 to 3.99)	
LDL (mg/dL)			0.95
<130	1.00 (Referent)	1.00 (Referent)	
>130	0.83 (0.41 to 1.68)	0.90 (0.37 to 2.21)	
HDL (mg/dL)			0.96
Normal	1.00 (Referent)	1.00 (Referent)	
Low^g^	1.65 (0.82 to 3.32)	1.81 (0.85 to 3.86)	
Age (1-year increment)	1.05 (1.02 to 1.08)	1.04 (1.00 to 1.08)	
Serum aspartate aminotransferase (IU/L)			—
<45	1.00 (Referent)	1.00 (Referent)	
>45	2.78 (1.13 to 6.89)	0.89 (0.25 to 3.15)	
Serum alanine aminotransferase (IU/L)			—
<45	1.00 (Referent)	1.00 (Referent)	
>45	1.17 (0.47 to 2.90)	2.38 (0.75 to 7.55)	
Serum platelet (109/L)			—
>150	1.00 (Referent)	1.00 (Referent)	
<150	1.37 (0.71 to 2.64)	3.50 (1.46 to 8.38)	
APRI index			—
<0.38	1.00 (Referent)	1.00 (Referent)	
>0.38	2.70 (1.35 to 5.42)	1.89 (0.71 to 5.00)	
FIB-4 index			—
<1.45	1.00 (Referent)	1.00 (Referent)	
>1.45	1.86 (0.82 to 4.22)	1.44 (0.57 to 3.67)	
Education			—
>9 years	1.00 (Referent)	1.00 (Referent)	
<9 years	0.90 (0.48 to 1.69)	2.91 (0.63 to 13.5)	
No formal education	1.38 (0.66 to 2.88)	3.09 (0.60 to 15.6)	
Cigarette smoking at enrollment			—
No	1.00 (Referent)	1.00 (Referent)	
Yes	1.13 (0.65 to 1.98)	2.07 (0.70 to 6.08)	
Habitual alcohol consumption^d^			—
No	1.00 (Referent)	1.00 (Referent)	
Yes	1.94 (1.10 to 3.40)	0.36 (0.05 to 2.83)	

a *P* value for the interaction term of metabolic related factors by sex. APRI = aspartate aminotransferase to platelet index; BMI = body mass index; CI = confidence interval; FIB-4 = fibrosis index; HBV/HCV = hepatitis B virus/hepatitis C virus; HCC = hepatocellular carcinoma; HDL = high-density lipoprotein; HR = hazard ratio; LDL = low-density lipoprotein; TG = serum triglyceride.

b Metabolic syndrome was defined as having at least 3 of the 5 components including central obesity (waist circumferences >90 cm for men and >80 cm for women), hypertriglycemia (TG > 150 mg/dL), low HDL cholesterol (<40 mg/dL in men and <50 mg/dL in women), hypertension (systolic blood pressure > 130 mmHg or diastolic blood pressure  > 80 mmHg or on medication for hypertension), and diabetes (fasting glucose level >126 mg/dL or on medication for diabetes).

c Metabolic score was defined as the number of metabolic factors included in metabolic syndrome.

d The adjusted hazard ratios for fasting glucose in interval scale and metabolic syndrome were separately modeled from that with categorized status of impaired fasting blood sugar. The estimated results for other variables were based on the model with categorized fasting blood sugar.

e The adjusted hazard ratios for BMI categories were separately modeled from that with BMI categories in group linear form.

f *P* value was based on interaction term of BMI using less than 23 as reference group by sex.

^g^HDL <40mg/dL in men and <50mg/dL in women

### Fasting Blood Sugar Level and Diabetic Status

The interaction terms for sex and fasting glucose level (*P* = .046) on HCC risk was statistically significant (Table 2). Compared with men with normal blood sugar, the HCC risk started to increase monotonously from prediabetic to diabetic (aHR = 2.10, 95% CI = 1.07 to 4.13) and 3.71 (95% CI = 2.01 to 6.86), respectively. In women, the adjusted hazard ratios for prediabetes and diabetes were 1.16 (95% CI = 0.48 to 2.83) and 1.47 (95% CI = 0.65 to 3.34), respectively. With every 10 mg/dl increase of fasting glucose level, a statistically significant 11% increased HCC risk (aHR = 1.11, 95% CI = 1.07 to 1.16) was found in men and was not statistically significant (aHR = 1.03, 95% CI = 0.97 to 1.10) in women. The adjusted men-to-women ratio of hazard ratios of developing HCC was 1.06 (95% CI = 0.99 to 1.14) with every 10 mg/dl increase of fasting glucose level (data not shown). The dose-response association between serum fasting glucose and HCC incidence was statistically significant in men (*P*_trend _< .001) but not in women (*P*_trend _= .34) (Table 2).

### Body Mass Index

The interaction terms for BMI (*P* = .03 for categorical and *P* = .05 for group linear) on HCC risk were statistically significant. Compared with BMI 23-25, underweight (BMI < 21, HR = 3.56, 95% CI = 1.18 to 10.8) and overweight (BMI > 25, HR = 3.81, 95% CI = 1.43 to 10.2) were associated with increased HCC risk in men; however, the relationship was linear with each advance of BMI category associated with a 36% higher risk of HCC in women (aHR = 1.36, 95% CI = 0.99 to 1.86). For women, the relationship between BMI and HCC risk was closer to linear trend (*P*_trend_ = .06). In men, it appeared as a U-shape relationship (*P*_trend_ = .80). The impact of higher BMI on increased HCC risk appears to be more pronounced in women than men, with men-to-women ratio of hazard ratio statistically significantly lower (adjusted ratio of HR'S  = 0.13, 95% CI = 0.02 to 0.86) when comparing BMI 23 to less than 25 with BMI less than 21 (data not shown).

### Other Metabolic Factors

For other metabolic factors including SBP, DBP, TG, TC, HDL, and LDL, the quartile grouping was not linearly associated with HCC and not modified by sex (Table 2).

### Gender Difference in HCC Predictors

In addition to metabolic factors, other statistically significant HCC predictors selected by the stepwise procedure stratified by sex are shown in Table 3. Older age, elevated AST, higher APRI, habitual alcohol consumption, prediabetes (HR = 1.89, 95% CI = 0.98 to 3.68), diabetes (HR = 3.44, 95% CI = 1.90 to 6.21), and every 10 mg/dl increase of fasting glucose level (HR = 1.11, 95% CI = 1.07 to 1.15) were statistically significant predictors for HCC in men. For women, the important predictors were older age (HR = 1.07, 95% CI = 1.04 to 1.10), higher BMI group (HR = 1.41, 95% CI = 1.07 to 1.87), elevated ALT levels (HR = 3.55, 95% CI = 1.62 to 7.75), and lower serum platelet count (HR = 5.01, 95% CI = 2.35 to 10.68).

**Table 3.** Stepwise selected multivariable analysis of 5 different metabolic factors and other risk factors associated with non-HBV/HCV–related HCC

Variables	Male^a^	Female^b^
	HR (95% CI)	HR (95% CI)
Metabolic syndrome	2.58 (1.22 to 5.48)	1.19 (0.58 to 2.45)
Metabolic score	1.77 (1.16 to 2.68)	1.28 (0.97 to 1.68)
Diabetes mellitus		
Normal	1.00 (Referent)	1.00 (Referent)
Prediabetes	1.89 (0.98 to 3.68)	1.30 (0.54 to 3.14)
Diabetes	3.44 (1.90 to 6.21)	1.70 (0.77 to 3.77)
Fasting glucose per 10 mg/dl increment	1.11 (1.07 to 1.15)	1.03 (0.97 to 1.10)
BMI (kg/m^2^)		
<21	3.23 (1.14 to 9.12)	—
21 to <23	2.54 (0.94 to 6.89)	—
23 to <25	1.00 (Referent)	—
25 to <27.3	3.07 (1.24 to 7.61)	—
>27.3	1.74 (0.61 to 4.53)	—
BMI, per advanced group		1.41 (1.07 to 1.87)
Age	1.07 (1.04 to 1.09)	1.07 (1.04 to 1.10)
Serum aspartate aminotransferase (IU/L)		
<45	1.00 (Referent)	—
>45	2.85 (1.42 to 5.70)	—
Serum alanine aminotransferase (IU/L)		
<45	—	1.00 (Referent)
>45	—	3.55 (1.62 to 7.75)
Serum platelet (10^9^/L)		
>150	—	1.00 (Referent)
<150	—	5.01 (2.35 to 10.68)
APRI index		
<0.38	1.00 (Referent)	—
>0.38	3.67 (2.06 to 6.55)	—
Habitual alcohol consumption		
No	1.00 (Referent)	—
Yes	2.06 (1.63 to 3.45)	—

a For men, the stepwise model selection process with categorized fasting blood sugar (normal, prediabetes, and diabetes) as main metabolic factor resulted in age, serum aspartate aminotransferase level, APRI index, and habitual alcohol consumption in the final model. All other selected metabolic factors including metabolic syndrome, metabolic score, fasting glucose per 10 mg/dl increment, and BMI categories were modeled separately with the aforementioned variables. APRI = aspartate aminotransferase to platelet index; BMI = body mass index; CI = confidence interval; FIB-4 = fibrosis index; HBV/HCV = hepatitis B virus/hepatitis C virus; HCC = hepatocellular carcinoma; HR = hazard ratio.

b For women, the stepwise model selection process with categorized fasting blood sugar (normal, prediabetes, and diabetes) as main metabolic factor resulted in age, serum alanine aminotransferase level, and serum platelet level in the final model. All other selected metabolic factors including metabolic syndrome, metabolic score, fasting glucose per 10 mg/dl increment, and BMI per advanced group were modeled separately with the aforementioned variables.

## Discussion

In this analysis specifically restricted to residents tested seronegative in HBsAg and anti-HCV, we found that both prediabetes and diabetes statistically significantly predicted the HCC risk in men only, with an 11% elevated HCC risk for every 10 mg/dl increment of fasting glucose. Sex was found to statistically significantly modify the association between fasting glucose (*P*_interaction_ = .046), BMI (*P*_interaction_ = .03), and HCC risk. The sex difference of BMI and HCC association comes from the U-shape relationship for men and the linear trend for women. In general, the association between fasting blood sugar and HCC tended to be stronger in men, whereas elevated BMI was associated with higher HCC risk in women than men. All other metabolic factors including SBP, DBP, TG, TC, HDL, and LDL were not associated with HCC and not modified by sex. In addition to metabolic factors, men and women had different HCC risk factors: elevated AST, APRI fibrosis index, and habitual alcohol consumption were related to HCC only in men; for women, increased ALT and lower platelet levels predicted HCC risk.

Most cohort studies demonstrated that diabetes was associated with an approximately 2-fold increased HCC risk (3). Among 5 studies stratified by sex (15-19), 4 showed higher risk in males (16-19), whereas 1 reported similar results in both males and females (15). Our finding of elevated HCC risk commencing from prediabetes status in men was consistent only with a large cohort study in Europe (19), with a significant interaction between glucose level and sex. A statistically significant dose-response trend of fasting glucose and HCC in men (HR = 1.16, 95% CI = 1.07 to 1.27 for fasting glucose level 110-125 mg/dL) was reported in a large Korean cohort study (17). Although women had the highest HCC risk (HR = 1.23, 95% CI = 1.00 to 1.55) at glucose level 110-125 mg/dL, the dose-response trend was not significant (17). Another large cohort study in Austria (20) reported a hazard ratio of 2.63 (95% CI = 1.15 to 5.99) with glucose level 5.3-6.0 mmol/l (95-109 mg/dL) in men, but no results were reported for women because of sparse cases (20).

The association between obesity and HCC consistently showed sex disparities (12,13). One meta-analysis (12) of 26 prospective studies with 25 337 HCC patients reported that obese men had higher risk than obese women (SRR = 1.91, 95% CI = 1.51 to 2.41 for men; SRR = 1.55, 95% CI = 1.30 to 1.85 for women), with a statistically significant interaction (*P* = .03). Another recent meta-analysis focusing on sex difference reported that HCC incidence increased faster in men than in women, and a nonlinear dose-response trend was noted for men (13). Our results show a U-shape relationship between BMI and HCC in men, with increased risk in either underweight (BMI < 21) or overweight (BMI > 25); however, a linear trend was found in women with a 41% increased risk per advanced BMI group. Yao et al. also found that the sex difference in obesity–liver cancer association was more pronounced in non-Asian studies (relevant risk [RR] = 2.31, 95% CI = 1.85 to 2.91, and RR = 1.56, 95% CI = 1.31 to 1.86) for men and women, respectively, with a significant interaction (*P*_interaction_ = .01). No such interaction was found in Asian studies. Our results were similar to the non-Asian studies because we excluded residents with HBV/HCV infections, whereas the Asian studies included in this meta-analysis by Yao et al. adjusted only for HBV/HCV infections.

Men were known to have more visceral fat than women, and it was suggested that visceral adiposity played a more important role in liver carcinogenesis than total adiposity (21,22). This body composition difference was thought to be driven by differing sex hormones, in that higher androgen receptor density increased visceral fat and estrogen promoted the accumulation of subcutaneous fat, which protects against inflammation (23). It was suggested that estrogens prevent HCC through inhibition of IL-6 expression by decreasing the activity of transcription factor, nuclear factor κB in Kupffer cells, and that, in turn, affects hepatocyte proliferation (10). Recently, Foxa1/a2 factors and their targets were proposed to be responsible for modulating sex hormones and produce gender disparities in HCC (24). Although the exact mechanism remains unclear, accumulating evidence highlights the regulatory roles of the estrogen and/or androgen receptors signaling in the key chromatin-modifying enzymes and metabolic microRNAs in the pathogenesis of both type 2 diabetes and HCC (25). Elevated fasting blood glucose was found to increase HCC risk independent of obesity in this analysis. This may explain why both obesity and insulin resistance affected HCC risk in men in our study. In addition to metabolic factors, we found that other risk factors also affected HCC risk differently between men and women. Elevated AST, APRI fibrosis index, and habitual alcohol consumption were related to HCC only in men. Because these three factors are closely related to alcohol, one possible explanation is that men are more susceptible to alcoholic liver cirrhosis. In women, increased ALT and lower platelet levels, which are biomarkers of liver cirrhosis, predicted HCC risk. Nevertheless, no previous study investigated HCC without HBV and/or HCV risk factors specifically focusing on gender difference, and further study is warranted.

Exclusion of HBV and HCV carriers provided us with a unique opportunity to avoid the modifying effects of viral hepatitis. However, occult HBV infection contributing to HCC risk cannot be overlooked in this HBV endemic area (26). It is not feasible to perform tests on this large cohort of generally healthy community residents to determine the occult HBV infection status, so the controlling or exclusion of occult HBV is not an option. The prevalence of occult HBV was not estimated in the general population, but according to previous studies in the same endemic areas, it should range between 3% (27) and 15% (28) in our population. Although the magnitude of association between diabetes and HCC looks similar to the association among HBV carriers, we found no association of obesity among HBV carriers in our previous study (4). In addition, the HCC incidence was compatible in our diabetic subjects to other diabetic cohorts in non-HBV endemic area (11.6 vs 4.1-13.4 /10^5^ person-years) (16). Therefore, although occult HBV may still play some role in the none-B-none-C HCC carcinogenesis, the chance that the entire association we observed was totally due to occult HBV should be minimal. Nevertheless, this study still provides some insight that there was a sex difference in the association between diabetes and obesity in relation to HCC occurrence. Hyperglycemia and HCC share several common risk factors, such as HCV infection, alcohol consumption, and obesity. Our study excluded viral hepatitis and collected detailed information on alcohol consumption, obesity, and lipid profile and adjusted them in the analysis. We examined fasting blood glucose instead of self-reported or secondary data from insurance claims to avoid underestimating diabetes prevalence and can further demonstrate whether the dose-response relationship is nonlinear. With a short follow-up period, it is possible that cancers appearing earlier after follow-up were present but undiagnosed at recruitment, causing the decrease of visceral adiposity and BMI, thus the possible reverse causation. There were 21 HCC cases diagnosed within 12 months after recruitment. We performed sensitivity analysis by excluding HCC cases diagnosed within the first year of follow-up and similar results were found (*P*_trend_ = .0081). Liver cirrhosis diagnosed at enrollment was also excluded, and the results remained the same (*P*_trend_ = .0003). This analysis was based on information at baseline, and the change of these metabolic factors may introduce misclassification. But there is no reason to believe that this misclassification is related to the occurrence of HCC; hence, the misclassification should be nondifferential and more likely to underestimate the true effect. More women attended this program because the screening dates were not set on weekends since it's a voluntary survey. Originally 60 237 women and 38 990 men were recruited, and because men had higher prevalence of HBV infection than women, 46 248 women (77%) and 28 534 (73%) men remained in final analysis. Because all the analyses were conducted separately by sex, the disproportion of sex should not affect the study outcome.

In conclusion, our study demonstrated that sex disparity exists in diabetes and obesity in relation to HCC. The elevated HCC risk starts from the glucose impairment stage in men, indicating that insulin-resistance-related hepatocarcinogenesis may occur years before diabetes diagnosis. The awareness of HCC risk with elevated blood glucose level and obesity may be helpful to promote early lifestyle modification or drug intervention. Whether good glycemic and weight control can reduce HCC risk warrants further investigation.

### Funding

NSC-94–2314-B002-268 from the National Science Council, Taipei, Taiwan.

### Notes

**Role of the funder:** The funding agency of this report had no role in the design, conduct, data collection, data analysis and interpretation as well as preparation and approval of this manuscript. The corresponding author had full access to all the data and had final responsibility in the decision to submit for publication.

**Conflicts of interest:** The authors declare that there are no conflicts of interest.

**Authors contributions:** MJK, CLC, WSY and HHC contributed to the design and execution of this work and all authors contributed to the interpretation of the results and the preparation of this manuscript. AMFY and HHC contributed to the recruitment of study participants and data collection. MJK, AMFY, HHC and CLC contributed to statistical analysis. WSY, JHK and PJC contributed to critical revision of the manuscript for important intellectual content. The final draft was approved by all authors.

**Previous presentations:** Abstract and partial contents of this manuscript were presented as a poster in the 24th annual meeting of the Asian Pacific Association for the Study of the Liver held in Istanbul, Turkey, 2015.

## Supplementary Material

pkaa036_Supplementary_Data
