# Supplementary material for: Gender Difference in the Association Between Metabolic Factors and Hepatocellular Carcinoma
Source: JNCI Cancer Spectr. 2020 May 8;4(5):pkaa036. doi: 10.1093/jncics/pkaa036 (PMC7583157; doi:10.1093/jncics/pkaa036)
Supplement: pkaa036_Supplementary_Data [file pkaa036_supplementary_data.pdf]

Supplementary Figure 1. The sex-specific dose-response association between metabolic factors and risk for HCC incidence

(A) Status of impaired fasting blood sugar

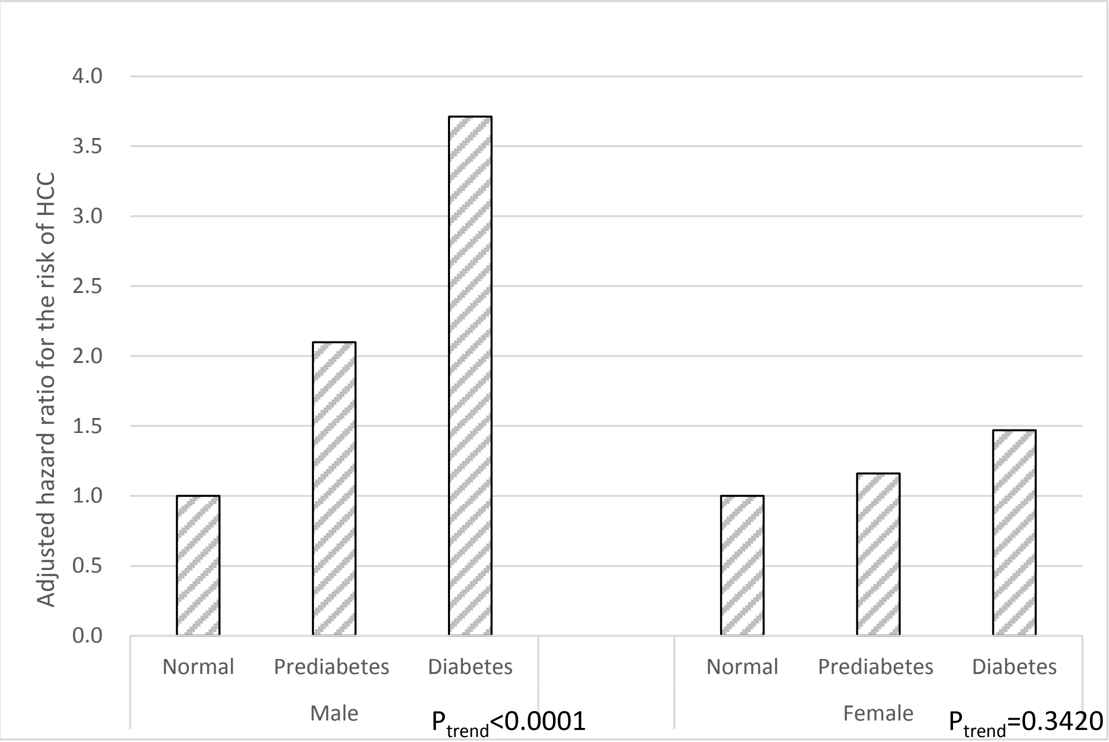

P value for interaction by sex = 0.0463

(B) BMI group

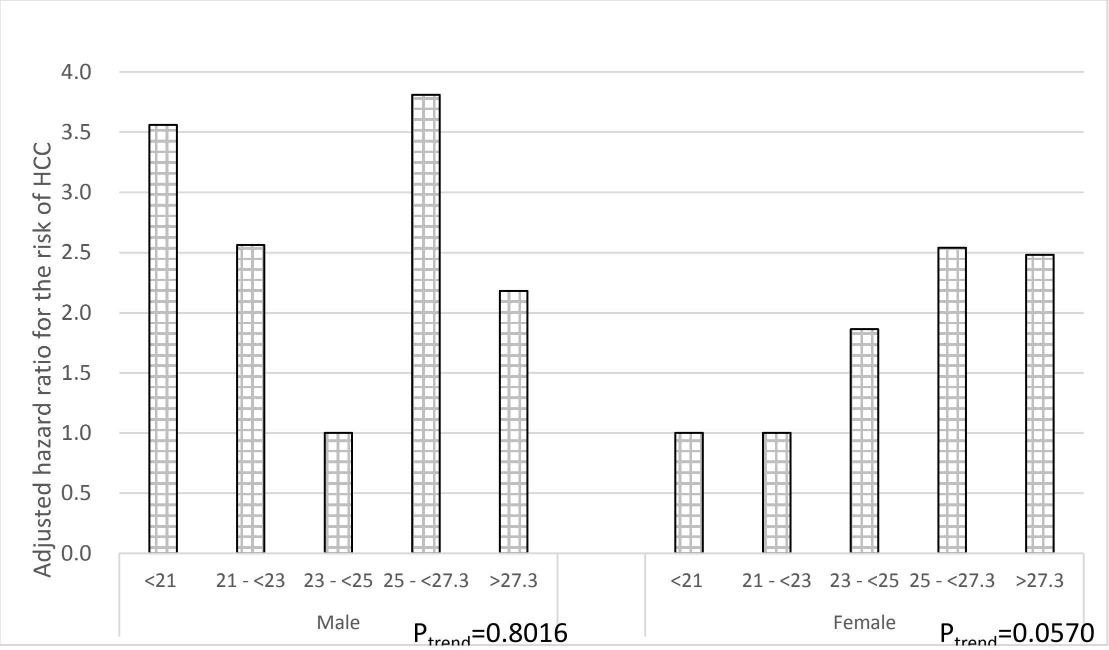

P value for interaction by sex = 0.0479

(C) SBP group

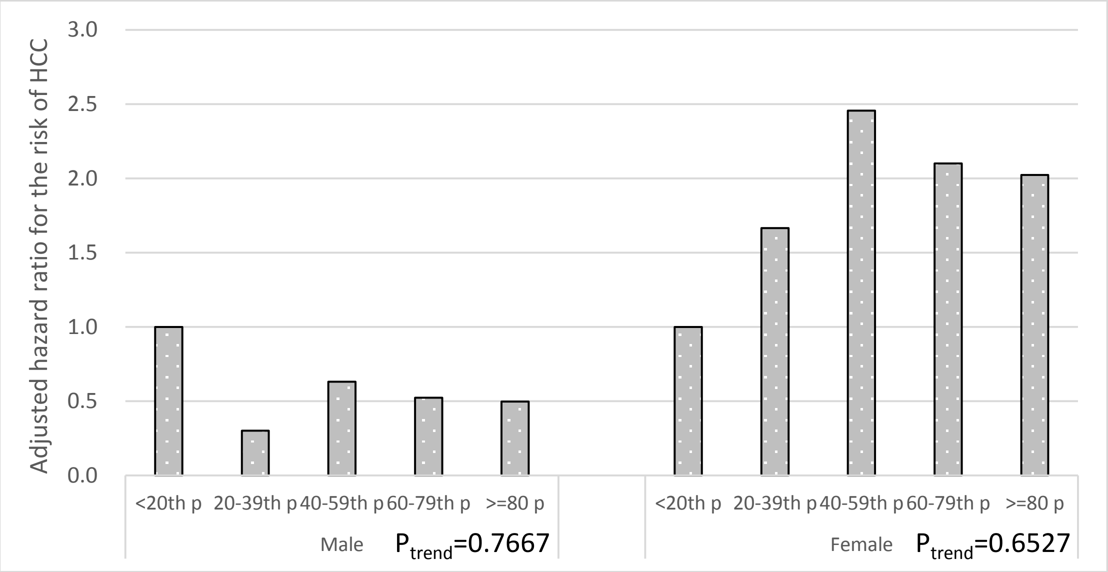

P value for interaction by sex = 0.4805

(D) DBP group

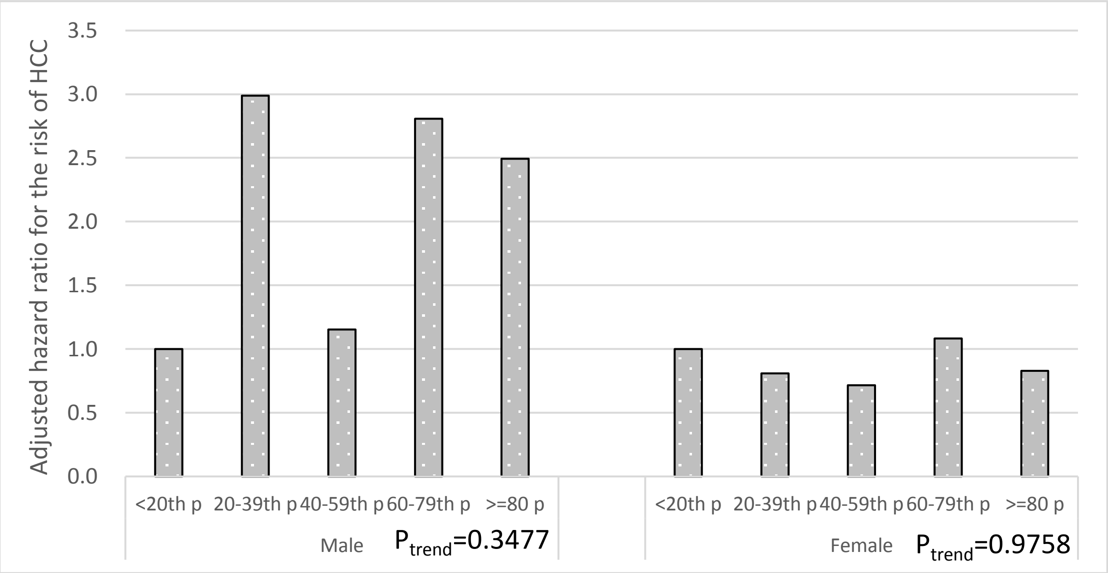

P value for interaction by sex = 0.5346

(E) TG group

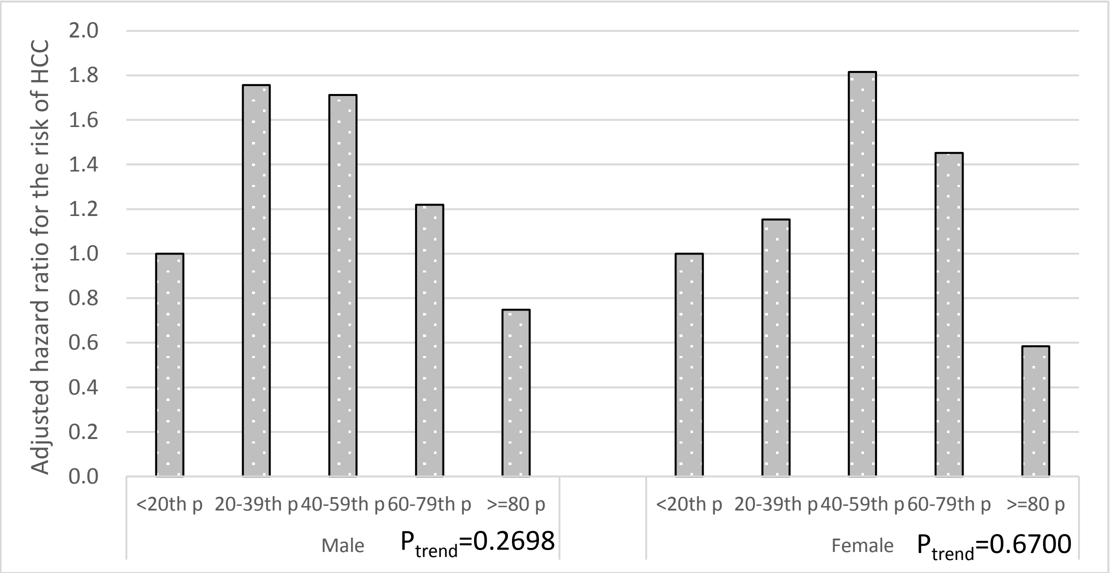

P value for interaction by sex = 0.4217

(F) TC group

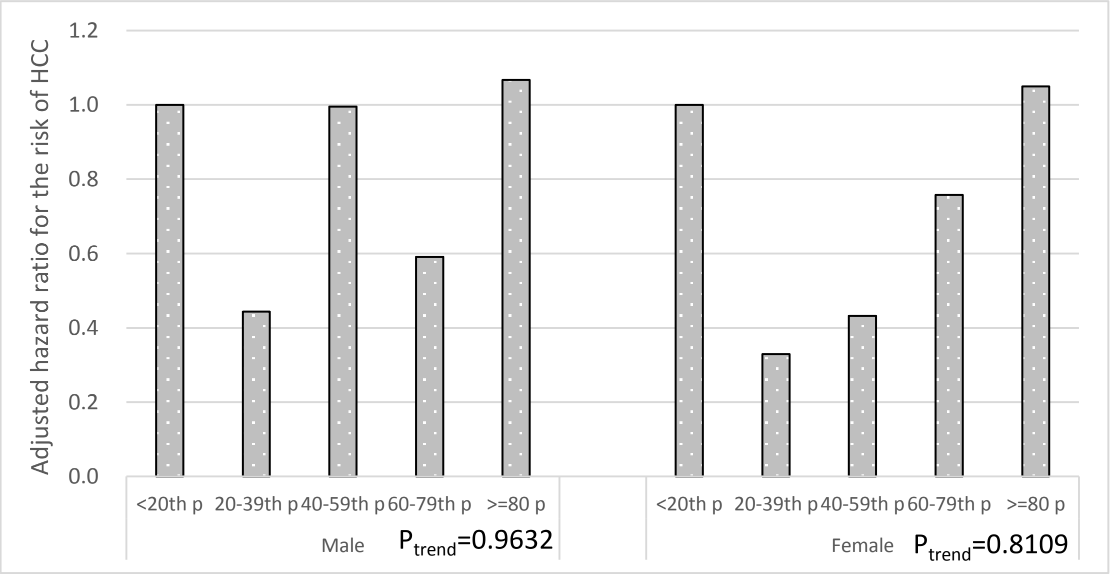

P value for interaction by sex = 0.6939

(G) HDL group

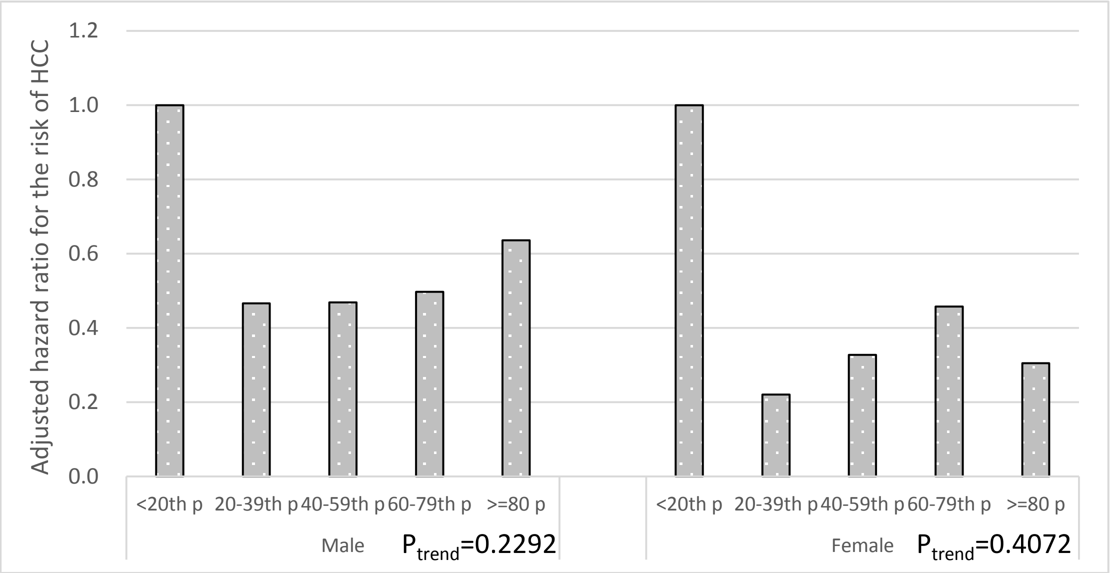

P value for interaction by sex = 0.4546

(H) LDL group

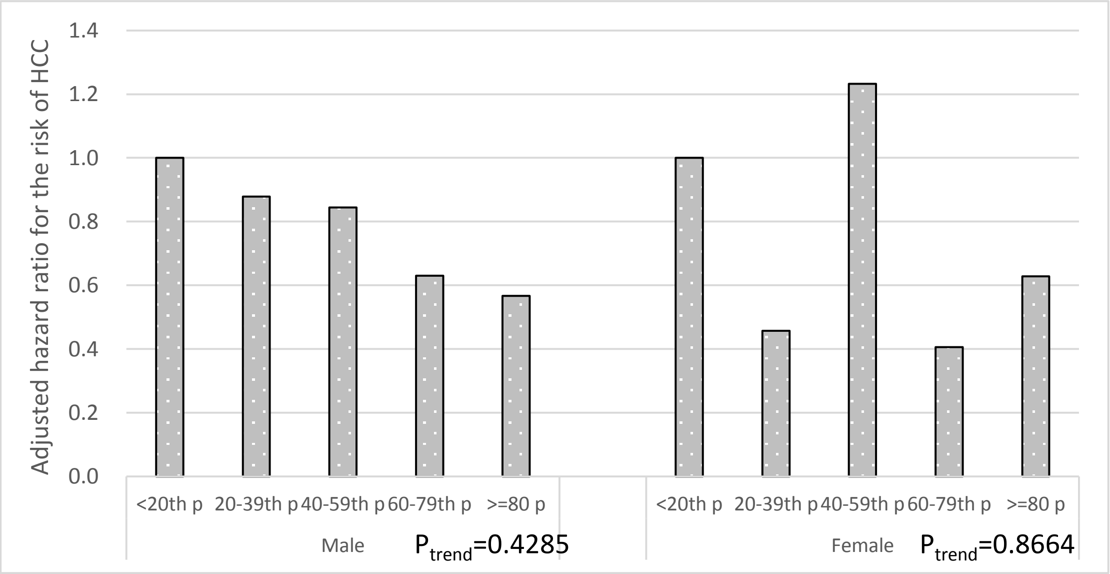

P value for interaction by sex = 0.4306
